# Management of Minor Traumatic Brain Injury in an ED Observation Unit

**DOI:** 10.5811/westjem.2021.4.50442

**Published:** 2021-07-15

**Authors:** Matthew A. Wheatley, Shikha Kapil, Amanda Lewis, Jessica Walsh O’Sullivan, Joshua Armentrout, Tim P. Moran, Anwar Osborne, Brooks L. Moore, Bryan Morse, Peter Rhee, Faiz Ahmad, Hany Atallah

**Affiliations:** * Emory University School of Medicine, Department of Emergency Medicine, Atlanta, Georgia; † Georgetown University School of Medicine, Department of Emergency Medicine, Washington, District of Columbia; ‡ Grady Health Systems, Department of Emergency Medicine, Atlanta, Georgia; § Atlanta Medical Center, Department of Emergency Medicine, Atlanta, Georgia; ¶ Maine Medical Center, Department of Surgery and Surgical Critical Care, Portland, Maine; || Westchester Medical Center, Department of Surgery, Trauma Surgery, and Surgical Critical Care, Valhalla, New York; # Emory University School of Medicine, Department of Neurosurgery, Atlanta, Georgia; ** Jackson Health System, Miami, Florida

## Abstract

**Introduction:**

Traumatic intracranial hemorrhages (TIH) have traditionally been managed in the intensive care unit (ICU) setting with neurosurgery consultation and repeat head CT (HCT) for each patient. Recent publications indicate patients with small TIH and normal neurological examinations who are not on anticoagulation do not require ICU-level care, repeat HCT, or neurosurgical consultation. It has been suggested that these patients can be safely discharged home after a short period of observation in emergency department observation units (EDOU) provided their symptoms do not progress.

**Methods:**

This study is a retrospective cross-sectional evaluation of an EDOU protocol for minor traumatic brain injury (mTBI). It was conducted at a Level I trauma center. The protocol was developed by emergency medicine, neurosurgery and trauma surgery and modeled after the Brain Injury Guidelines (BIG). All patients were managed by attendings in the ED with discretionary neurosurgery and trauma surgery consultations. Patients were eligible for the mTBI protocol if they met BIG 1 or BIG 2 criteria (no intoxication, no anticoagulation, normal neurological examination, no or non-displaced skull fracture, subdural or intraparenchymal hematoma up to 7 millimeters, trace to localized subarachnoid hemorrhage), and had no other injuries or medical co-morbidities requiring admission. Protocol in the EDOU included routine neurological checks, symptom management, and repeat HCT for progression of symptoms. The EDOU group was compared with historical controls admitted with primary diagnosis of TIH over the 12 months prior to the initiation of the mTBI protocols. Primary outcome was reduction in EDOU length of stay (LOS) as compared to inpatient LOS. Secondary outcomes included rates of neurosurgical consultation, repeat HCT, conversion to inpatient admission, and need for emergent neurosurgical intervention.

**Results:**

There were 169 patients placed on the mTBI protocol between September 1, 2016 and August 31, 2019. The control group consisted of 53 inpatients. Median LOS (interquartile range [IQR]) for EDOU patients was 24.8 (IQR: 18.8 – 29.9) hours compared with a median LOS for the comparison group of 60.2 (IQR: 45.1 – 85.0) hours (P < .001). In the EDOU group 47 (27.8%) patients got a repeat HCT compared with 40 (75.5%) inpatients, and 106 (62.7%) had a neurosurgical consultation compared with 53 (100%) inpatients. Subdural hematoma was the most common type of hemorrhage. It was found in 60 (35.5%) patients, and subarachnoid hemorrhage was found in 56 cases (33.1%). Eleven patients had multicompartment hemorrhage of various classifications. Twelve (7.1%) patients required hospital admission from the EDOU. None of the EDOU patients required emergent neurosurgical intervention.

**Conclusion:**

Patients with minor TIH can be managed in an EDOU using an mTBI protocol and discretionary neurosurgical consults and repeat HCT. This is associated with a significant reduction in length of stay.

## INTRODUCTION

Traumatic brain injury (TBI) is a frequent cause for emergency department (ED) visits. The US Centers for Disease Control and Prevention (CDC) estimated there were 2.5 million ED visits related to TBI in 2013, which represents an increase from 2007.^1^ Traumatic brain injury is grossly classified as mild, moderate, and severe based on the presenting Glasgow Coma Scale (GCS) score with mild TBI (mTBI) defined as a GCS of 13–15.^2^

Clinical policies and decision tools exist to aid the emergency physician (EP) in deciding which patients with mTBI need brain imaging.^3^,^4^ Once traumatic intracranial hemorrhages (TIH) are identified with head computed tomography (HCT), patients are typically admitted or transferred to a trauma center with neurosurgical capabilities. This can happen regardless of the size and location of the hemorrhage, or clinical condition of the patient. Inpatient care is typically in an intensive care unit (ICU) setting so that they can be monitored closely for clinical deterioration. In addition, patients routinely receive repeat HCT and neurosurgical consultation.^5^

Recent studies show routine follow-up HCT in many patients are not predictive of the need for neurosurgical intervention and this practice should be reserved for patients who demonstrate deterioration of neurologic exam.^6^–^9^ Retrospective studies by Joseph et al have concluded that minor TIH patients have low risk of requiring neurosurgical intervention and, therefore, can be managed without neurosurgical consultation.^10^,^11^ Multiple studies have examined the necessity of ICU admission for minor TIH. Patients with isolated traumatic subarachnoid hemorrhage have low rates of clinical and radiographic deterioration.^12^–^14^ Other studies have suggested that patients with minor TIH largely do not receive critical care interventions and, therefore, do not benefit from ICU admission.^15^,^16^ These are retrospective analyses with no universal definition of minor TIH. Hence, the question has come up about using ED observation units (EDOU) to monitor patients with minor TIH.^14^,^17^

In their 2015 validation of the Brian Injury Guideline (BIG) protocol, Joseph et al recommended up to 24-hour observation for patients with minor TIH without repeat HCT or neurosurgical consultation.^18^ Minor TIH fits with other conditions commonly managed in the EDOU setting, as it is a single condition and patients can be managed in under 24 hours.^19^ This allows the visits to be more focused, which leads to decreased length of stay (LOS) and decreased healthcare costs.^20^–^27^ Randomized controlled trials (RCT) that have compared EDOU and inpatient care for conditions such as chest pain, asthma, atrial fibrillation, and transient ischemic attack have found EDOU care to be more efficient and cost effective.^28^–^36^ Yun and colleagues have looked at managing patients with TIH in an EDOU setting where they performed a retrospective analysis of TIH patients before and after an EDOU protocol was implemented.^37^ They reported that use of the protocol was associated with decreased need for admission and lower likelihood of worsening TIH on repeat CT. There was no difference in LOS in EDOU patients pre-protocol and during the protocol.

This study evaluates the outcomes of patients managed in the EDOU using an mTBI protocol based on BIG criteria.

## METHODS

This is a retrospective cross-sectional study performed at a Level I trauma center. Initial workup in the acute phase of care was provided primarily by the emergency medicine (EM) team consisting of an EM attending and either an EM resident or an EM advanced practice provider. Here, the trauma team was either activated to co-manage patients based on pre-set protocols or consulted at the discretion of the EM attending.

The EDOU mTBI protocol was created by a multidisciplinary team of physicians from the trauma surgery service, EM, and neurosurgery. The EDOU protocol was based on the BIG protocol.^11^,^18^ We altered the protocol slightly to exclude epidural hematomas based on institutional expert opinion. This practice change was implemented as a quality improvement project first piloted September 1–December 31, 2016. In this phase, patients who met BIG 1 criteria (Table 1) were eligible for the EDOU protocol. Trauma and neurosurgical consultations were required for each patient. Beginning January 1, 2017, patients who met BIG 1 or 2 criteria were permitted in the EDOU. Trauma and neurosurgical consultations were at the discretion of the EM attending in all phases of care. Patients who were unable to ambulate independently, had intractable pain or vomiting, or other significant traumatic injuries were considered ineligible for EDOU. The guidelines for this protocol are summarized in Table 2.

Interventions in the EDOU consisted of neurologic checks every two hours for up to 23 hours. These standard assessments, performed by nursing, involve testing for level of alertness, orientation, and gross deficits in limbs. Evidence of decreased mental status, seizure, or focal neurologic deficit prompted an emergent repeat HCT and consultation with both trauma surgery and neurosurgery. Symptoms were controlled with antiemetics and analgesics as needed. In the absence of clinical deterioration, repeat HCT was ordered at the discretion of the EDOU team. Patients were discharged home if symptoms were controlled with oral medication and they were able to eat and perform activities of daily living unassisted. Patients who were unable to do this were converted to inpatient status. They were admitted to the trauma service if they needed further treatment for their head injuries. Some were admitted to internal medicine due to occult medical issues that were identified during observation.

The intervention group was identified through an EDOU census report generated through the electronic health record (EHR). Because the EHR allowed use of the discrete variable “EDOU Pathway” it was not necessary to use *International Classification of Diseases, 10^th^ Modification* (ICD-10) codes to identify all the patients in the EDOU on this pathway. The database was queried for all patients on the mTBI protocol from its inception on September 1, 2016, through August 31, 2019. The report provides patient level ED and EDOU LOS data as well as final disposition: inpatient conversion or discharge from EDOU. Trained chart abstractors (EM residents) obtained age, gender, mechanism of injury, initial HCT reading by radiologist, TIH category as determined by trauma surgeons, disposition from the EDOU (be it admission or discharge to home), and follow-up information. Length of stay for the intervention group was calculated on the EHR report unless specified below. We defined ED LOS as patient arrival until they physically left the department. Length of stay in the EDOU was calculated as time of arrival in the EDOU until the time of the admission or discharge order in the EHR. Admission and discharge order times were manually abstracted via chart review. Total LOS was calculated as the sum of ED and EDOU LOS.

The comparison group was made up of patients admitted to the trauma service for TIH from September 1, 2015–August 31, 2016. Patients were identified by querying the trauma registry for all patients who were admitted with a primary diagnosis of TIH based on ICD-10 code. The trauma registry is a database maintained by the Trauma and Acute Care Surgery service. Minor TBI inclusion criteria were retrospectively applied to these patients to select the group that would have been eligible for EDOU. Trained chart abstractors obtained demographic, imaging, disposition, and follow-up information on comparison group patients. Although the group for comparison was derived from the registry database at our institution and the intervention group was derived from an EHR report, ultimately the chart abstractors used the same EHR system (Epic Systems Corporation, Verona, WI) to obtain the results used in the analyses.

We described LOS using medians and interquartile ranges. All other variables were described using counts and percentages. The primary research question regarded whether the mTBI protocol reduced the median LOS. This was tested using quantile regressions. Quantile evaluates the association between some predictor and a given quantile/percentile of the outcome while controlling for other variables (eg, whether an intervention reduces the 50^th^ percentile/median or 75^th^ percentile of an outcome). Adjusted analyses controlled for the effects of age, gender, mechanism of injury, neurosurgery consultation, repeat HCT, and BIG level. We computed *P*-values and 95% confidence intervals (CI) as bootstrapped estimates (10,000 resamples). Categorical patient characteristics were compared across groups using the χ^2^ test. Analyses were conducted using R v. 3.5.2 (R Foundation for Statistical Computing, Vienna, Austria).

## RESULTS

During the study period 209 patients were placed on the mTBI protocol. We excluded 40 patients from this analysis because they did not have an acute TIH or were admitted as inpatients to the trauma service but boarding in the EDOU. The control group consisted of 53 patients. Demographic and clinical information for the intervention and comparison groups are summarized in Table 3.

**Figure 1:**
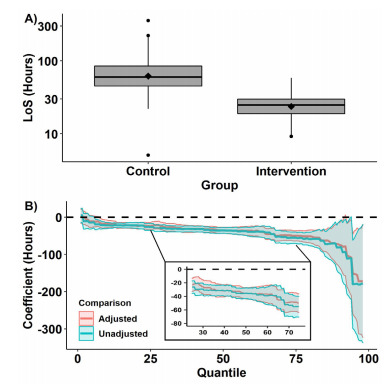
A) A box and whisker plot depicting length of stay as a function of intervention group. The solid lines within the boxes depict the median for each group and the diamonds within the boxes depict the means for each group. Note that the data are presented on a log10 scale. B) The results of the quantile regressions evaluating the association between the protocols and length of stay. The solid lines depict the difference between the intervention and control groups (eg, the median/50th percentile for the intervention group was approximately 35 hours shorter than for the control group; however, the 75th percentile was approximately 55 hours shorter for the intervention group than for the control group). Negative coefficients indicate that the intervention group had reduced lengths of stay relative to the control group. Shaded regions depict the 95% confidence intervals. The inset section of panel B highlights the change in cost between the 25th and 75th percentiles *LOS*, length of stay.

**Figure 2:**
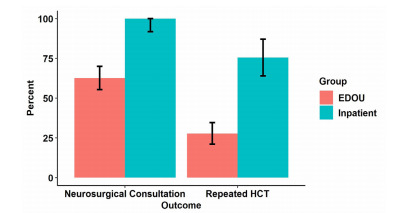
Graphic representation of difference in neurosurgical consultation and repeat head computed tomography between intervention (EDOU) and control (Inpatient) groups. *HCT*, head computed tomography; *EDOU*, emergency department observation unit.

The primary outcome is presented in Figure 1. Median LOS (IQR) for EDOU patients was 24.8 (IQR: 18.8 – 29.9) hours compared with a median LOS for the comparison group of 60.2 (IQR: 45.1 – 85.0) hours. This 35.4 (95% CI, 27.3 – 43.5) hour reduction was significant (*P* < .001). In the adjusted analyses, the intervention was associated with a 35.5 (95% CI, 27.2 – 43.8, *P* < .001) hour reduction is LOS. In the EDOU group 47 (27.8%) patients got a repeat HCT compared with 40 (75.5%) inpatients, and 106 (62.7%) had a neurosurgical consultation compared with 53 (100%) inpatients (Figure 2). Subdural hematoma was the most common type of hemorrhage. It was found in 60 (35.5%) of patients, and subarachnoid hemorrhage was found in 56 cases (33.1%). Eleven patients had multicompartment hemorrhage of various classifications.

Twelve (7.1%) patients required hospital admission from the EDOU. Reasons for admission are explained in Table 4. Average inpatient LOS was 3.25 days. Only three patients required ICU care, and four were admitted to the internal medicine service. Ten of the admitted patients were able to be discharged home following their hospitalization. One patient was transferred to hospice, and one was discharged to rehab. None of the patients managed in the EDOU required neurosurgical intervention. There was only one patient death in the EDOU group. Based on review of clinical records, this was thought to be due to metabolic encephalopathy and not head injury.

Follow-up information was available on only 45 (26.6%) patients. Twelve patients reported mild symptoms of headache or dizziness. One patient had persistent headache three months later. No patients required readmission or neurosurgical intervention due to their head injuries. Two patients were called back to the ED due to CT over-reads. Neither of these visits resulted in an admission. Seven patients received outpatient imaging due to persistent symptoms, but no neurosurgical intervention was required for these patients.

## DISCUSSION

We found a significant difference in our primary outcome of EDOU vs inpatient LOS. Management of patients with mTBI in the EDOU was associated with significant reduction in LOS when compared with patients in inpatient settings. This is consistent with the above studies on EDOU vs inpatient care. This finding differs somewhat from the EDOU study by Yun et al in that they did not compare EDOU and inpatient data, but rather the LOS in the ED portion of care only.^37^ This difference is not as surprising as the preponderance of other studies showing benefit in LOS for EDOU pathways when compared to usual care in an inpatient setting.

Overall, our protocol is similar to the one reported in the Yun study. There were minor differences in inclusion criteria such as the upper limit of subdural hematoma. Interventions in the EDOU were similar between the two groups including frequent neurologic checks and repeat HCT for clinical deterioration. In addition, we found a low rate of adverse events in the EDOU group, which is consistent with previous studies on minor TIH. None of the patients in the intervention group required emergent neurosurgical intervention. The most common reasons for inpatient admission were persistent symptoms due to head injury or other traumatic or medical issues that presented during the observation period. This is summarized in Table 4. Further study is needed to determine predictors for inpatient conversion in this group.

Patients in the EDOU had a lower rate of neurosurgical consultation and repeat HCT when compared with their inpatient counterparts. Repeat HCTs were ordered based on clinical concern or recommendations from radiology or neurosurgical consultants. Further study is needed to determine the clinical necessity of these interventions in the EDOU setting.

## LIMITATIONS

There are many limitations to this study given its single-center, retrospective design. A large, multicenter RCT is needed to better understand the true relationship between EDOU care and LOS. In addition, because adverse outcomes in BIG 1 and 2 class TIH are rare, larger numbers are needed to truly understand the safety of this approach. However, because TIH patient are a high-risk population a more precise understanding of the rates of hemorrhage progression and need for emergent neurosurgical intervention is essential before EDOU care can be widely recommended.

The biggest limitation of this study is the limited follow-up information in the intervention group. Because this study began as a quality improvement initiative, there initially was not a robust mechanism to conduct follow-up interviews to investigate whether patients were still experiencing symptoms or had repeated medical visits due to their injuries. This is an important area for future study. Patients were chosen for the EDOU based on clinician gestalt that the patient fit within the inclusion/exclusion guidelines. This could introduce bias into the results as patients who were thought to be sicker or more complicated were likely admitted to inpatient units. The control group for this study is small and thus may limit the strength of association of some of the outcomes. This study was conducted in an urban teaching facility and Level 1 trauma center; thus, it may not be translatable to smaller or rural centers without trauma or neurosurgical services. Further studies involving non-Level I trauma centers are necessary.

## CONCLUSION

Use of an EDOU to observe patients with minor traumatic hemorrhage as defined by the Brain Injury Guidelines classification was associated with significantly reduced length of stay and low overall incidence of adverse events. Care in the ED observation unit was also associated with fewer repeat head computed tomography and neurosurgical consultations. Further study is needed to determine predictors for inpatient conversion, follow-up needs, and ability of smaller, non-trauma centers to use this protocol.

**Population Health Research Capsule**

What do we already know about this issue?

*Patients with small traumatic intracranial hemorrhages (TIH) often utilize intensive care, serial head computed tomographies (CT) and neurosurgical consultation, even though they rarely benefit from these resources*.

What was the research question?

*Can management of patients with minor traumatic intracranial hemorrhages be accomplished in emergency department observational units (EDOUs) and use fewer resources?*

What was the major finding of the study?

*Minor TIH patients in EDOUs are associated with a shorter length of stay, fewer repeat CTs and neurosurgical consults*.

How does this improve population health?

*Stable patients with small traumatic hemorrhages may not benefit from more interventions and critical care. This could lead to cost savings for this group of patients*.

## Figures and Tables

**Table 1:** Traumatic intracranial hemorrhage classification based on Brain Injury Guidelines (BIG).^11^

	BIG 1	BIG 2	BIG 3
Neurological examination findings	Normal	Normal	Normal or abnormal
Intoxication	No	No	Yes
Anticoagulation	No	No	Yes
Skull fracture	No	Nondisplaced	Displaced
SDH,	≤ 4 mm	5–7 mm	≥ 8 mm
EDH, mm	No	No	Any size
IPH	≤ 4 mm, 1 location	5–7 mm, 2 locations	≥ 8 mm, multiple locations
SAH	Trace	Localized	Scattered
IVH	No	No	Yes

*SDH*, subdural hematoma; *mm*, millimeters; *EDH*, epidural hematoma; *IPH*, intraparenchymal hemorrhage; *SAH*, subarachnoid hemorrhage; *IVH*, intraventricular hemorrhage.

**Table 2:** Emergency department observation unit guidelines for patients with minor traumatic brain injury.

EDOU transfer criteria
Meets Brain Injury Guideline (BIG) 1 or BIG 2 criteria
Patient has spine cleared or is in Aspen collar and is able to ambulate without assistance
No other traumatic injuries that need continued evaluation or treatment. Splinted extremities are acceptable provided the patient is able to ambulate
Patient not having intractable pain/vomiting
Stable vital signs
Consultation in ED by trauma surgery and neurological surgery teams as deemed appropriate by ED attending
Exclusion criteria
Not meeting all of BIG 1 or BIG 2 criteria
Other injuries that still need evaluation/treatment
Inability to ambulate
Intractable pain/vomiting
Unstable vital signs (persistent tachycardia; tachypnea; hypotension)
Other indications for admission
Potential interventions
Serial neurologic exams including vital signs every 2 hours
6–23 hour observation for change in neurological status
Advance diet as tolerated
Antiemetics/analgesics as needed
Repeat CT as indicated
Decision points/acute interventions
STAT repeat CT head and call to neurosurgery and trauma residents on call for
Decreased mental status based on Q2 hour checks
Seizure at any point
New focal neurologic deficits found on neuro checks
STAT trauma evaluation for:
Development of abnormal vital signs
Intractable pain
Inability to ambulate
Discharge criteria
Home
Acceptable vital signs
Normal serial neurologic exams
Tolerating diet as they were prior to admission
Able to ambulate and perform activities of daily living without assistance
Admit
Deterioration in clinical condition
Development of any exclusion criteria – including over read of initial CT head that includes BIG 2 or 3 criteria

*ED*, emergency department; *CT*, computed tomography.

**Table 3:** Patient characteristics.

Characteristic	Control (n = 53)	Intervention (n = 169)	P
Age	36 (26.5 – 55)	41 (27.5 – 57)	.39
Gender			.34
Male	35 (66.0)	98 (58.0)	
Female	18 (34.0)	71 (42.0)	
Mechanism			.08
Assault	15 (28.3)	26 (15.4)	
Bike/ATV/Scooter	1 (1.9)	8 (4.7)	
Fall	10 (18.9)	57 (33.7)	
MVC	20 (37.7)	67 (39.6)	
Ped vs Vehicle	4 (7.5)	7 (4.1)	
Other	3 (5.7)	4 (2.4)	
Big Protocol			.40
1	41 (77.4)	135 (79.9)	
2	12 (22.6)	30 (17.8)	
3	0 (0)	4 (2.4)	
NSGY	53 (100)	106 (62.7)	<.001
Repeat HCT	40 (75.5)	46 (27.4)	<.001
LOS	60.2 (45.1 – 85.0)	24.8 (18.8 – 29.9)	<.001

*ATV*, all terrain vehicle; *MVC*, motor-vehicle collision; *Ped*, pedestrian; *NSGY*, neurosurgery; *HCT*, head computed tomography; *LOS*, length of stay.

**Table 4:** Patients admitted following emergency department observation unit observation period.

Patient number	Age/gender	HCT finding	Reason for admission	Type of bed	Inpatient LOS
15	25/F	Trace SAH (overread as negative)	Persistent tachycardia	Trauma floor	2 days
16	59/M	Subacute subdural	Dizziness, bradycardia	Medical telemetry	4 days
17	25/F	Trace SAH	Vomiting, worsening CT	Trauma ICU	2 days
22	31/F	Trace SAH vs artifact	Pain control	Trauma floor	3 days
58	40/M	Subdural skull fracture	Worsening CT	Trauma ICU	5 days
107	51/M	Scattered punctate hyperdensities likely artifact	Persistent Confusion	Trauma floor	6 days
108	79/F	4mm SDH	Gait instability	Trauma floor	2 days
114	77/M	3mm SDH	Worsening mental status	Medical ICU	11 days
115	77/M	Small SAH vs artifact	New atrial flutter	Medical telemetry	1 day
119	90/F	Trace SAH	Unable to ambulate	Medical floor	1 day
133	27/F	Streak artifact vs hemorrhagic contusion	Dizziness	Trauma floor	1 day
134	18/F	R frontal SAH, R IPH	CT over-read	Trauma floor	1 day

*HCT*, head computed tomography; *LOS*, length of stay; *M*, male; *F*, female; *SAH*, subarachnoid hemorrhage; *mm*, millimeters; *ICU*, intensive care unit; *SDH*, subdural hematoma; *IPH*, intraparenchymal hemorrhage.
